# Systolic blood pressure and recurrent stroke in patients with different lesion patterns on diffusion weighted imaging

**DOI:** 10.1111/jch.14543

**Published:** 2022-08-12

**Authors:** Pan Chen, Qiong Wu, Xuewei Xie, Jing Jing, Hongqiu Gu, Xianwei Wang, Xia Meng, Liping Liu, Yilong Wang, Yongjun Wang, the CHANCE Investigators

**Affiliations:** ^1^ Department of Neurology Beijing Tiantan Hospital Capital Medical University Beijing China; ^2^ China National Clinical Research Center for Neurological Diseases Beijing China; ^3^ Advanced Innovation Center for Human Brain Protection Capital Medical University Beijing China; ^4^ Research Unit of Artificial Intelligence in Cerebrovascular Disease Chinese Academy of Medical Sciences Beijing China; ^5^ Department of Neurology The Second Hospital of Dalian Medical University Dalian China

**Keywords:** blood pressure, diffusion‐weighted imaging, ischemic stroke, transient ischemic attack

## Abstract

Little is known about the relationship between baseline systolic blood pressure (SBP) and subsequent clinical events in patients with different lesion patterns on diffusion weighted imaging (DWI). We analyzed the Acute Non‐disabling Cerebrovascular Events (CHANCE) trial dataset. Patients were categorized into negative DW imaging (no detectable lesions), lacunar infarction (single lesion ≤15 mm) and non‐lacunar infarction (single lesion >15 mm or multiple lesions) based on lesion patterns on DWI. The primary outcome was recurrent stroke within 90 days. Cox proportional hazards models were used to assess the association between SBP levels and stroke outcomes in patients with different lesion patterns. A total of 1089 patients were analyzed. We found 258 cases (23.7%) with negative DW imaging, 392 (36.0%) with lacunar infarction and 439 (40.3%) with non‐lacunar infarction. Patients with non‐lacunar infarction had the highest incidence of stroke at 90‐day (*P* < .001). In non‐lacunar infarction group, compared with SBP < 160 mmHg, patients with SBP ≥ 160 mmHg had significantly higher risk of 90‐day recurrent stroke (20.3% vs. 10.7%; adjusted hazard ratio 1.81, 95% confidence interval 1.09–3.00). No significant association was found between SBP and clinical outcomes in patients with negative DWI and lacunar stroke groups. The result at 1 year was similar as at 90‐day. Therefore, non‐lacunar infarction, the most common lesion pattern in CHANCE study, had the highest risk of recurrent stroke and combined vascular events both in 90 days and 1 year. High baseline SBP was significantly associated with increased risk of short‐ and long‐term recurrent strokes in patients with non‐lacunar infarction.

## 1 INTRODUCTION

The risk of stroke recurrence after minor stroke and transient ischemic attack (TIA) has improved significantly over the past few years as treatment has improved.^1^ But minor stroke and TIA still have a high recurrence rate.^2^ Hypertension is a powerful but controllable risk factor for the incident and recurrent stroke.^3^, ^4^, ^5^ In acute phase of ischemic stroke, sustained high BP may result in increased infarct growth and an unfavorable outcome.^6^ It is recommended that patients with blood pressure (BP) ≥160/100 mmHg should be promptly treated to control BP.^3^ However, it is also important to maintain ample organ perfusion with less stringent BP targets to avoid hypotension.^7^ Lower blood pressure has an adverse effect on the recovery of the ischemic semidark band.^8^, ^9^ In a word, the effect of baseline BP on stroke prognosis is still highly controversial.

Nowadays, acute ischemic stroke is considered to be a heterogeneous disease with different pathogenesis,^4^ which further complicates the relationship between hypertension and stroke prognosis. A report has suggested that the potential mechanisms by which elevated baseline BP affects stroke subtype may be different.^10^ Whether baseline blood pressure affects prognosis by influencing stroke subtype is unclear. Currently, the identification of ischemic stroke subtype relies on clinical evaluation supported by various diagnostic studies, advances in stroke imaging makes it possible to develop criteria for the most likely mechanism behind the ischemic stroke.^5^, ^11^ As an important part of multimodal magnetic resonance imaging (MRI), diffusion weighted imaging (DWI) has been shown to accurately diagnose the ischemic state of stroke to determine the mechanism of stroke occurrence and to assess the prognosis of stroke.^11^, ^12^

Thus, in this study, we hypothesized that the effects of baseline SBP on clinical outcomes depended on the ischemic lesion patterns characterized by DWI features. The aim of the current study was to assess the relationship between SBP and clinical outcomes in patients with negative DW imaging, lacunar infarction and non‐lacunar infarction using data from the Clopidogrel in High‐Risk Patients with Acute Non‐disabling Cerebrovascular Events (CHANCE) trial.

## 2 METHODS

### 2.1 Study design

This study was based on the CHANCE trial. The study design and main results of the trial have been published previously.^13^ Brief, this was a randomized, double‐blind, placebo‐controlled clinical trial conducted at 114 clinical sites in China to test the efficacy and safety of dual antiplatelet (clopidogrel plus aspirin) versus mono antiplatelet (aspirin alone) initiated within 24 h of symptom onset on reducing the risk of any stroke (ischemic or hemorrhagic) during 90 days follow up in high‐risk patients with acute minor stroke (NIH Stroke Scale [NIHSS] ≤3) or TIA (ABCD2 ≥4). Patients with presumed cardiac source of embolus, such as atrial fibrillation or prosthetic cardiac valve were excluded in CHANCE trial.

The CHANCE protocol was approved by the ethics committee of Beijing Tiantan hospital as well as all other participating clinical centers. Written informed consent was obtained from all participants or their legal proxies.

### 2.2 Data collection

Data on demographic characteristics, life style risk factors, and medical history were collected through face to face interviews by trained interviews. Three blood pressure were recorded at the time when the patients were randomly allocated to groups by trained physicians. At admission, blood pressure was measured from the left arm in the supine position within 24 h after symptoms ictus. Three blood pressure readings separated by at least two minutes were recorded by doctors or trained nurses. The average of three readings was defined as the blood pressure on admission and used in our analysis.

### 2.3 Patients selection, image analysis and interpretation

Recruited patients who underwent baseline (within 7 days of symptom onset) magnetic resonance examinations (3.0 or 1.5 tesla) at baseline with the T1‐weighted imaging, T2‐weighted imaging, and DWI sequences were analyzed in this subgroup study. Those without baseline MRI or without any of the sequences as mentioned above were excluded from the study.

All MRIs were collected from individual sites in digital format and were read in MRI Reading Center by 2 readers (Xinying Zou and Jing Jing) who were blinded to patients’ baseline and outcome information. They assessed all scans for acute ischemic lesions, according to predefined criteria for identifying ischemic lesions on neuroimaging as follows. Acute infarcts on DWI were diagnosed when these lesions were shown to be hyperintense on the DWI. Uninterrupted lesions visible in contiguous territories were considered as a single lesion. In this analysis, patients were divided into three groups based on their brain MRI findings: negative DW imaging was the group of patients without any DWI lesions. Lacunar infarction was defined as a single lesion with the largest diameter of ≤15 mm in an axial slice of DWI for penetrating artery infarction of the basal ganglia, corona radiate, thalamus, or pons. Non‐lacunar infarction was defined as a single lesions with the largest diameter of >15 mm or multiple lesions in an axial slice of DWI.

### 2.4 Clinical outcomes

Neurologic examination, physical evaluation, and brain imaging were performed if a patient had transient or persistent neurologic deterioration during follow‐up. The primary efficacy outcome was a total stroke (ischemic or hemorrhagic) within 90 days.^13^ The secondary efficacy outcome included combined vascular events (ischemic stroke, hemorrhagic stroke, myocardial infarction, or vascular death) within 90 days, and stroke recurrence and combined vascular events during a 1‐year follow‐up period. The safety outcome was any bleeding event, as per the Global Utilization of Streptokinase and Tissue Plasminogen Activator for Occluded Coronary Arteries (GUSTO) definition.^14^ Patients were first evaluated by certified investigators who were blinded to patients’ clinical status and treatment allocation. The definitions of ischemic stroke, composite vascular event, and bleeding were consistent with previously reported outcomes of the CHANCE trial.^13^ All reported efficacy and safety outcomes were further confirmed by a central adjudication committee that was blinded to the study group assignments.

### 2.5 Statistical analysis

Continuous variables were presented as mean with SD or median with interquartile range, and categorical variables were reported as percentages. According to the ischemic lesion patterns, the baseline characteristics were compared in the patients with SBP ≥160 mmHg and <160 mmHg using Student's *t*‐test or Kruskal‐Wallis test for continuous variables, and Chi‐square tests or Fisher exact test for categorical variables. Additionally, a sensitivity analysis was performed in the patients with SBP ≥140 mmHg and <140 mmHg, because of 140 mmHg is a common cutoff for SBP. Cox proportional hazards regression was used to estimate the association between SBP and risk of study outcomes by calculating the hazard ratios (HRs) and 95% confidence intervals (CIs). The potential covariates such as age, sex, body mass index, current or previous smoking, antiplatelet treatment, medical history of stroke, TIA, hypertension and hyperlipidemia were included in the multivariate models. All *P*‐values were 2‐tailed, and a significance level of .05 was used. Statistical analyses were performed using SAS Version 9.4 software (SAS Institute, Cary, NC).

## 3 RESULTS

### 3.1 Patient demographics and baseline characteristics

Overall, 5170 patients were recruited to CHANCE between October 2009 and July 2012, 1089 patients at 45 sites undergoing MRI at baseline with all the sequences as demanded were included in this subgroup analysis.

Baseline characteristics of the patients included in this study and all others in the CHANCE trial were basically similar, except that patients included in this subgroup analysis had lower body mass index levels, lower prevalence of a history of ischemic stroke/TIA, and have higher admission NIHSS score (Supplemental Table SI). Characteristics of the study population, according to acute cerebral infarcts subtype, are shown in Table 1. Among the 1089 patients included in the current subgroup analysis, non‐lacunar infarction was observed in 40.3% (439/1089), lacunar infarction in 36.0% (392/1089), and negative DW imaging in 23.7% (258/1089) of patients. In patients with non‐lacunar infarction, those with SBP ≥160 mmHg had higher levels of body mass index.

**TABLE 1:** Baseline characteristics of patients with non‐lacunar infarction, lacunar infarction and negative DW imaging in the current subgroup analysis of the CHANCE trial

	Non‐lacunar infarction			Lacunar infarction			Negative DW imaging		
	High SBP^a^	Low SBP		High SBP	Low SBP		High SBP	Low SBP	
	*N* = 177	*N* = 262	*P*‐value	*N* = 182	*N* = 210	*P*‐value	*N* = 80	*N* = 178	*P*‐value
Age, mean (SD), y	63 (11)	63 (11)	.89	63 (11)	62 (10)	.14	66 (10)	63 (11)	.06
Male, *n* (%)	112 (63.3)	178 (67.9)	.31	117 (64.3)	150 (71.4)	.13	49 (61.3)	107 (60.1)	.86
Current/previous smoking, *n* (%)	70 (39.5)	117 (44.7)	.29	80 (44.0)	102 (48.6)	.36	29 (36.3)	60 (33.7)	.69
Current/ previous drinking, *n* (%)	60 (33.9)	83 (31.7)	.63	64 (35.2)	62 (29.5)	.23	23 (28.8)	43 (24.2)	.43
Body mass index, mean (SD), kg/m^2^	24.7 (3.5)	23.9 (3.1)	.01	24.6 (3.3)	24.5 (3.0)	.8	24.5 (3.1)	24.4 (3.2)	.82
Time to randomization			.74			.42			.86
<6h	32 (18.1)	41 (15.6)		20 (11.0)	32 (15.2)		18 (22.5)	45 (25.3)	
6–12 h	56 (31.6)	95 (36.3)		54 (29.7)	62 (29.5)		27 (33.8)	56 (31.5)	
12–18 h	34 (19.2)	45 (17.2)		43 (23.6)	38 (18.1)		17 (21.3)	32 (18.0)	
≥18 h	55 (31.1)	81 (30.9)		65 (35.7)	78 (37.1)		18 (22.5)	45 (25.3)	
Admission NIHSS score, median (IQR)	2 (1‐3)	2 (1‐3)	.81	2 (1‐2)	2 (1‐3)	.48	0(0‐1)	0 (0‐2)	.33
Medical history, *n* (%)									
Ischemic stroke/TIA	33 (18.6)	53 (20.2)	.68	35 (19.2)	36 (17.1)	.59	22 (27.5)	34 (19.1)	.13
Myocardial infarction	3 (1.7)	9 (3.4)	.27	3 (1.6)	4 (1.9)	.85	0 (.0)	0 (.0)	NA
Atrial fibrillation	6 (3.4)	8 (3.1)	.84	2 (1.1)	1 (.5)	.48	2 (2.5)	2 (1.1)	.41
Heart Failure	7 (4.0)	5 (1.9)	.19	1 (.5)	0 (.0)	.28	1 (1.3)	5 (2.8)	.44
Hypertension	128 (72.3)	154 (58.8)	.004	130 (71.4)	121 (57.6)	.005	62 (77.5)	115 (64.6)	.04
Diabetes mellitus	37 (20.9)	64 (24.4)	.39	34 (18.7)	45 (21.4)	.50	12 (15.0)	35 (19.7)	.37
Hyperlipidemia	21 (11.9)	31 (11.8)	.99	15 (8.2)	27 (12.9)	.14	8 (10.0)	35 (19.7)	.05
Antiplatelet treatment			.80			.59			.65
Clopidogrel + Aspirin	92 (52.0)	133 (50.8)		86 (47.3)	105 (50.0)		34 (42.5)	81 (45.5)	
Aspirin only	85 (48.0)	129 (49.2)		96 (52.7)	105 (50.0)		46 (57.5)	97 (54.5)	

Abbreviations: DWI, diffusion weighted imaging; IQR, interquartile range; NA, not applicable; NIHSS, National Institute of Health Stroke Scale; SD, standard deviation; TIA, transient ischemic attack.

^a^ High SBP means baseline SBP value ≥160 mmHg and low SBP means baseline SBP value < 160 mmHg.

### 3.2 Efficacy outcomes

**FIGURE 1:**
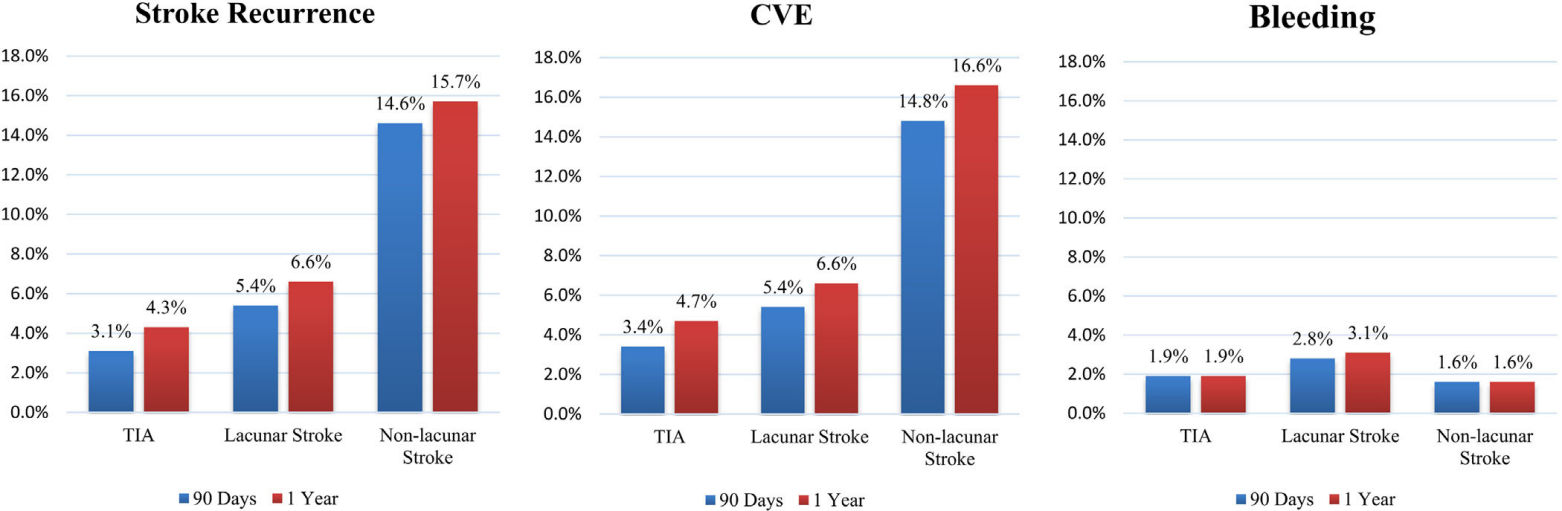
Crude event rates in patients with different lesion patterns on DWI. CVE, combined vascular events

During 90 days of follow‐up, a total of 93 (8.5%) patients with stroke recurrence and 95 (8.7%) with combined vascular events were identified. Among different subtypes stroke recurrence (14.6% in non‐lacunar infarction, 5.4% in lacunar infarction, and 3.1% in negative DW imaging group, *P *< .001) and combined vascular events risk (14.8% in non‐lacunar infarction group, 5.4% in lacunar infarction, and 3.4% in negative DW imaging group, *P *< .001) were highest in non‐lacunar infarction group (Figure 1). After adjusting for age, sex, medical history, admission NHISS score, and other covariates, compared to SBP <160 mmHg, SBP ≥160 mmHg was significantly associated with increased risk of recurrent stroke (20.3% vs. 10.7%; HR 1.81, 95% CI 1.09–3.00; *P* = .02) in patients with non‐lacunar infarction, while no significant association was observed among patients with lacunar infarction (5.7% vs. 4.9%; HR .84, 95% CI .34–2.06; *P* = .71) or negative DW imaging (2.8% vs. 3.8%; HR .99, 95% CI .19–5.20; *P* = .86) (Table 2). In addition, compared to patients with SBP <160 mmHg, those with SBP ≥160 mmHg had significant increased risk of combined vascular events in non‐lacunar infarction group (20.9% vs. 10.7%; HR 1.85, 95% CI 1.12–3.07; *P* = .02). Similarly, higher SBP at baseline could independently predict recurrence of stroke among patients with non‐lacunar infarction (20.9% vs. 12.2%; HR 1.65, 95% CI 1.01–2.67; *P* = .04) after 1‐year follow‐up (Table 3). In a study with 140 mmHg as the intercept point, the risk of recurrent stroke remained highest in patients with non‐lacunar infarction on DWI, but after adjusting for age, sex, medical history, admission NHISS score, and other covariates, the SBP <140 mmHg group and the SBP No significant differences were found in the risk of recurrent stroke, combined vascular events, or bleeding between the SBP <140 mmHg and SBP ≥140 mmHg groups (Results are presented in the Supplemental Table SII).

**TABLE 2:** Effects of baseline systolic blood pressure on 90‐day efficacy and safety outcomes based on stroke subtypes

Subgroup		Crude event rates no. (%)		Crude		Multivariable adjusted	
Outcome	No. of patients	High SBP^a^	Low SBP	HR (95% CI)	*P‐*value	HR (95% CI)	*P‐*value
Non‐lacunar infarction	439						
Stroke recurrence		36 (20.3)	28 (10.7)	1.94 (1.18‐3.18)	.009	1.81 (1.09‐3.00)	.02
Combined vascular events		37 (20.9)	28 (10.7)	2.00 (1.22‐3.26)	.006	1.85 (1.12‐3.07)	.02
Bleeding		1 (.6)	6 (2.3)	.30 (.04‐2.58)	.27	.39 (.04‐3.64)	.41
Lacunar infarction	392						
Stroke recurrence		9 (4.9)	12 (5.7)	.87 (.37‐2.06)	.75	.84 (.34‐2.06)	.71
Combined vascular events		9 (4.9)	12 (5.7)	.87 (.37‐2.06)	.75	.84 (.34‐2.06)	.71
Bleeding		5 (2.7)	6 (2.9)	.96 (.29‐3.15)	.95	1.85 (1.12‐3.07)	.02
Negative DW imaging	258						
Stroke recurrence		3 (3.8)	5 (2.8)	1.34 (.32‐5.60)	.69	.99 (.19‐5.20)	.86
Combined vascular events		3 (3.8)	6 (3.4)	1.11 (.28‐4.45)	.88	.86 (.18‐4.20)	.86
Bleeding		1 (1.3)	4 (2.2)	.55 (.06‐4.95)	.60	.76 (.08‐7.48)	.82

Abbreviations: CI, confidence interval; DWI, diffusion weighted imaging.; HR, hazard ratio.

Adjusted for age, sex, body mass index, current or previous smoking, medical history of stroke, TIA, hypertension and hyperlipidemia treatment, and randomization group.

^a^ High SBP means baseline SBP value ≥160 mmHg and low SBP means baseline SBP value <160 mmHg.

**TABLE 3:** Effects of baseline systolic blood pressure on 1‐year efficacy and safety outcomes based on stroke subtypes

Subgroup		Crude event rates no. (%)		Crude		Multivariable adjusted	
Outcome	No. of patients	High SBP^a^	Low SBP	HR (95% CI)	*P‐*value	HR (95% CI)	*P‐*value
Non‐lacunar infarction	439						
Stroke recurrence		37 (20.9)	32 (12.2)	1.76 (1.10‐2.83)	.02	1.65 (1.01‐2.67)	.04
Combined vascular events		38 (21.5)	35 (13.4)	1.66 (1.05‐2.64)	.037	1.55 (.97‐2.49)	.07
Bleeding		1 (.6)	6 (2.3)	.25 (.03‐2.04)	.19	.28 (.03‐2.34)	.25
Lacunar infarction	392						
Stroke recurrence		12 (6.6)	14 (6.7)	.99 (.46‐2.14)	.98	1.01 (.46‐2.24)	.98
Combined vascular events		12 (6.6)	14 (6.7)	.99 (.46‐2.14)	.98	1.01 (.46‐2.24)	.98
Bleeding		6 (3.3)	6 (2.9)	1.17 (.38‐3.62)	.79	1.10 (.34‐3.54)	.87
Negative DW imaging	258						
Stroke recurrence		3 (3.8)	8 (4.5)	.84 (.22‐3.17)	.80	.69 (.16‐2.93)	.61
Combined vascular events		3 (3.8)	9 (5.1)	.74 (.20‐2.75)	.66	.63 (.15‐2.58)	.52
Bleeding		1 (1.3)	4 (2.2)	.56 (.06‐4.98)	.60	.77 (.08‐7.58)	.82

Abbreviations: CI, confidence interval; DWI, diffusion weighted imaging.; HR, hazard ratio.

Adjusted for age, sex, body mass index, current or previous smoking, medical history of stroke, TIA, hypertension and hyperlipidemia treatment, and randomization group.

^a^ High SBP means baseline SBP value ≥160 mmHg and low SBP means baseline SBP value <160 mmHg.

### 3.3 Safety outcomes

At 90 days, 7 (1.6%), 11 (2.8%), and 5 (1.9%) occurred bleeding events in patients with non‐lacunar infarction, lacunar infarction and negative DW imaging, respectively. When came to 1 year, we found, 7 (1.6%), 12 (3.1%), and 5 (1.9%) non‐lacunar infarction, lacunar infarction, and negative DW imaging participants encountered bleeding events. For incidence of any bleeding event, no significant difference was found between patients with SBP ≥160 mmHg and SBP <160 mmHg among different ischemic lesion patterns at 90 days or 1 year (Tables 2 and 3). Similar conclusions were drawn in studies with 140 mmHg as cutoff point.

## 4 DISCUSSION

In this subgroup analysis of the CHANCE trial, we found that patients with non‐lacunar infarction on DWI had the highest risk of recurrent stroke. Among non‐lacunar infarction patients, baseline SBP ≥160 mmHg indicated a higher risk of poor clinical outcomes at 90 days. There were no significant associations between baseline SBP level and stroke outcomes in negative DW imaging and lacunar infarction patients. The results from a 1‐year follow‐up were almost consistent with 90 days’ findings.

The finding of patients with non‐lacunar infarction had the highest risk of poor clinical events have important implications for targeting secondary stroke prevention. In CHANCE study, patients with cardiac source embolism were excluded. The etiological diagnosis for non‐lacunar infarction in this subgroup study was mainly artery to artery embolic strokes.^13^, ^15^ It was consistent with previous findings.^16^, ^17^ BP levels are an important factor in embolic instability,^18^ and patients with stroke and TIA were at high risk of early recurrent stroke, usually due to arterial thromboembolism.^19^, ^20^ Although previous investigators were inclined to define ischemic stroke subtypes by Trial of Org 10172 in Acute Stroke Treatment (TOAST) classification, which needed a series of examinations and increased the complexity of clinical decision‐making.^21^ Our data indicate the use of a new imaging based diagnostic method for the evaluation of patients presenting with an acute minor stroke.

Another finding of this subgroup is non‐lacunar infarction individuals with baseline SBP ≥160 mmHg was full in hazard compared with baseline SBP <160 mmHg. High baseline SBP almost doubled the risk of recurrence stroke and CVE which indicated elevated SBP as a good predictor for poor clinical outcomes in the non‐lacunar infarction population. However, at a threshold of 140 mmHg, there was no significant difference in the risk of recurrent stroke, combined vascular events and bleeding between the SBP <140 mmHg and SBP <140 mmHg groups. The small sample size in group of SBP <140 mmHg might affect the result. The relationship between blood pressure and stroke recurrence in patients with stroke has been studied a lot, but significant controversy exists.^22^, ^23^ On the one hand, higher blood pressure may be associated with recurrent stroke and death. Studies have shown that intensive blood pressure control may be effective to prevent the risk of hemorrhagic stroke in patients with a history of ischemic stroke.^24^ A 5 mmHg reduction in systolic blood pressure (SBP) from 120 mmHg to ≥170 mmHg is associated with an approximately 10% reduction in the risk of major cardiovascular events.^25^ On the other hand, hypoperfusion caused by low blood pressure may also lead to the progression of stroke.^26^ The effect of immediate hypotensive therapy after acute ischemic stroke has been studied in China Antihypertensive Trial in Acute Ischemic Stroke (CATIS), it's finding that early antihypertensive therapy was associated with a reduced rate of stroke recurrence in patients with a history of hypertension.^27^ This is consistent with our results, especially for non‐lacunar infarction patients.

The underlying mechanism can explain the relationship between baseline SBP and clinical outcomes in non‐lacunar infarction may as follows. Firstly, baseline high blood pressure might be an indicator of the previous history of hypertension. Thus the high risk may due to the effects of elevated blood pressure on the progression of atherosclerosis.^28^, ^29^, ^30^ Secondly, high SBP is a predictor of ulceration of carotid plaques which are associated with a high risk of ischemic stroke.^31^ Thirdly, since the etiological diagnosis for non‐lacunar infarction in this subgroup study was mainly artery to artery embolic strokes, multiple acute cerebral infarcts in non‐lacunar infarction may indicate an unstable source of thromboembolism.^32^ In the first few days after TIA and minor stroke onset, the underlying atherosclerotic plaque is most unstable and the risk of recurrence is highest. Blood pressure can affect the blood flow velocity and shear stress over unstable embolus. The research we were based on the subgroups of the Third China National Stroke Registry III found that large artery stenosis and infarction number were independent predictors of 1‐year stroke recurrence in low‐moderate risk but not in high‐risk patients with TIA or MIS stratified by ABCD2 score.^33^ This also highlights the importance of early detection and intervention of stroke.

No significant association was found between baseline SBP level and stroke outcomes in lacunar infarction or negative DW imaging patients. The result indicated high blood pressure plays a different role on stroke recurrence in respective of stroke subtype. The SPS3 trial showed that the lowing of SBP to a target of less than 130 mmHg in patients with recent lacunar infarction resulted in non‐significant reductions in all stroke, disabling or fatal stroke, and major vascular events, and a significant reduction in intracerebral stroke.^34^ The CATIS trial which included about 20% lacunar infarction also showed antihypertension did not reduce the likelihood of death and major disability at 14 days or hospital discharge.^35^ The ischemic lesion pattern is one of the factors should be taken into consideration in controlling blood pressure for the secondary prevention of stroke. In future, the clinical trial of blood pressure lowering within 24 h after symptom onset may exclude lacunar infarction and negative DW imaging patients.

This study has several limitations. Firstly, our observational study used data from a randomized control trial and therefore, is potentially subject to bias. For example, the CHANCE study only included acute minor stroke patients who were defined by a score of 3 or less at the time of randomization on the National Institutes of Health Stroke Scale or TIA. Thus, our findings may not apply to other ischemic stroke patients. Secondary, we only described the association between baseline SBP and clinical outcomes in different stroke pattern. Some other hemodynamic measures like diastolic blood pressure, pulse pressure and mean arterial pressure may also be associated with a poor outcome. A further analysis is in need.

In conclusion, patients with non‐lacunar infarction on DWI and together with high SBP at baseline are at higher risk for both early and late poor outcome events. Urgent initiation of effective blood pressure management as well as other treatments is of importance to those patients.

## CONFLICTS OF INTEREST

The authors declare that they have no competing interests.

## AUTHOR CONTRIBUTION

Yongjun Wang designed the study. Xuewei Xie, Pan Chen and Qiong Wu interpreted analysis of the data and prepared the report. Zixiao Li, Yilong Wang, Hongqiu Gu, Liping Liu, and Hao Li contributed to comments on the draft manuscript and revised the report. Xia Meng coordinated the study and revised the report. Hongqiu Gu conducted the statistical analysis. Jing Jing and Xianwei Wang contributed to comments on the draft manuscript and revised the report.

## Supporting information

Supporting Information.

## References

[1] Shahjouei S , Sadighi A , Chaudhary D , et al. A 5‐decade analysis of incidence trends of ischemic stroke after transient ischemic attack: a systematic review and meta‐analysis. JAMA Neurol. 2021;78(1):77‐87. 33044505 10.1001/jamaneurol.2020.3627 PMC7551236

[2] Amarenco P , Lavallee PC , Labreuche J , et al. One‐year risk of stroke after transient ischemic attack or minor stroke. N Engl J Med. 2016;374(16):1533‐1542. 27096581 10.1056/NEJMoa1412981

[3] Whelton PK , Carey RM , Aronow WS , et al. 2017 ACC/AHA/AAPA/ABC/ACPM/AGS/APhA/ASH/ASPC/NMA/PCNA guideline for the prevention, detection, evaluation, and management of high blood pressure in adults: a report of the American college of cardiology/American heart association task force on clinical practice guidelines. J Am Coll Cardiol. 2017;71(19):e127‐e248. 29146535 10.1016/j.jacc.2017.11.006

[4] Smith M , Reddy U , Robba C , Sharma D , Citerio G . Acute ischaemic stroke: challenges for the intensivist. Intensive Care Med. 2019;45(9):1177‐1189. 31346678 10.1007/s00134-019-05705-y

[5] Ko Y , Lee S , Chung JW , et al. MRI‐based algorithm for acute ischemic stroke subtype classification. J Stroke. 2014;16(3):161‐172. 25328874 10.5853/jos.2014.16.3.161 PMC4200592

[6] Wahlgren N , Ahmed N , Eriksson N , et al. Multivariable analysis of outcome predictors and adjustment of main outcome results to baseline data profile in randomized controlled trials: safe implementation of thrombolysis in stroke‐monitoring study (SITS‐MOST). Stroke. 2008;39(12):3316‐3322. 18927461 10.1161/STROKEAHA.107.510768

[7] Lin MP , Ovbiagele B , Markovic D , Towfighi A . Systolic blood pressure and mortality after stroke: too low, no go? Stroke. 2015;46(5):1307‐1313. 25765723 10.1161/STROKEAHA.115.008821

[8] Puhr‐Westerheide D , Tiedt S , Rotkopf LT , et al. Clinical and imaging parameters associated with hyperacute infarction growth in large vessel occlusion stroke. Stroke. 2019;50(10):2799‐2804. 31426729 10.1161/STROKEAHA.119.025809

[9] Arenillas JF , Cortijo E , García‐Bermejo P , et al. Relative cerebral blood volume is associated with collateral status and infarct growth in stroke patients in SWIFT PRIME. J Cereb Blood Flow Metab. 2018;38(10):1839‐1847. 29135347 10.1177/0271678X17740293 PMC6168913

[10] Sandset EC , Jusufovic M , Sandset PM , Bath PM , Berge E . Effects of blood pressure‐lowering treatment in different subtypes of acute ischemic stroke. Stroke. 2015;46(3):877‐879. 25657183 10.1161/STROKEAHA.114.008512

[11] Hurford R , Li L , Lovett N , et al. Prognostic value of “tissue‐based” definitions of TIA and minor stroke: population‐based study. Neurology. 2019;92(21):e2455‐e2461. 30996061 10.1212/WNL.0000000000007531 PMC6541432

[12] Kim BJ , Kang HG , Kim HJ , et al. Magnetic resonance imaging in acute ischemic stroke treatment. J Stroke. 2014;16(3):131‐145. 25328872 10.5853/jos.2014.16.3.131 PMC4200598

[13] Wang Y , Wang Y , Zhao X , et al. Clopidogrel with aspirin in acute minor stroke or transient ischemic attack. N Engl J Med. 2013;369(1):11‐19. 23803136 10.1056/NEJMoa1215340

[14] An international randomized trial comparing four thrombolytic strategies for acute myocardial infarction. The GUSTO investigators. N Engl J Med. 1993;329(10):673‐682. 8204123 10.1056/NEJM199309023291001

[15] Ritz K , Denswil NP , Stam OC , van Lieshout JJ , Daemen MJ . Cause and mechanisms of intracranial atherosclerosis. Circulation. 2014;130(16):1407‐1414. 25311618 10.1161/CIRCULATIONAHA.114.011147

[16] Veltkamp R , Pearce LA , Korompoki E , et al. Characteristics of recurrent ischemic stroke after embolic stroke of undetermined source: secondary analysis of a randomized clinical trial. JAMA Neurol. 2020;77(10):1233‐1240. 32628266 10.1001/jamaneurol.2020.1995 PMC7550970

[17] Kauw F , Takx RAP , de Jong H , Velthuis BK , Kappelle LJ , Dankbaar JW . Clinical and imaging predictors of recurrent ischemic stroke: a systematic review and meta‐analysis. Cerebrovasc Dis. 2018;45(5‐6):279‐287. 29936515 10.1159/000490422 PMC6492524

[18] Higuchi E , Toi S , Shirai Y , et al. Prevalence of microembolic signals in embolic stroke of undetermined source and other subtypes of ischemic stroke. Stroke. 2020;51(2):655‐658. 31771457 10.1161/STROKEAHA.119.027008

[19] Hankey GJ . Dual antiplatelet therapy in acute transient ischemic attack and minor stroke. N Engl J Med. 2013;369(1):82‐83. 23803138 10.1056/NEJMe1305127

[20] Campbell BCV , Khatri P . Stroke. The Lancet. 2020;396(10244):129‐142. 10.1016/S0140-6736(20)31179-X 32653056

[21] Ghandehari K . Comments on stroke subtypes classifications. Int J Stroke. 2010;5(6):514‐516. 10.1111/j.1747-4949.2010.00524.x 21050411

[22] Appleton JP , Sprigg N , Bath PM . Blood pressure management in acute stroke. BMJ. 2016;1(2):72‐82. 10.1136/svn-2016-000020 PMC5435190 28959467

[23] McManus M , Liebeskind DS . Blood pressure in acute ischemic stroke. J Clin Neurol. 2016;12(2):137‐146. 26833984 10.3988/jcn.2016.12.2.137 PMC4828558

[24] Kitagawa K , Arima H , Yamamoto Y , et al. Intensive or standard blood pressure control in patients with a history of ischemic stroke: RESPECT post hoc analysis. Hypertens Res. 2022;45(4):591‐601. 35241817 10.1038/s41440-022-00862-y

[25] Rahimi K , Bidel Z , Nazarzadeh M , et al. Pharmacological blood pressure lowering for primary and secondary prevention of cardiovascular disease across different levels of blood pressure: an individual participant‐level data meta‐analysis. The Lancet. 2021;397(10285):1625‐1636. 10.1016/S0140-6736(21)00590-0 PMC8102467 33933205

[26] Gasecki D , Kwarciany M , Kowalczyk K , Narkiewicz K , Karaszewski B . Blood pressure management in acute ischemic stroke. Curr Hypertens Rep. 2020;23(1):3. 33305339 10.1007/s11906-020-01120-7 PMC7728631

[27] Zhang R , Zhong C , Zhang Y , et al. Immediate antihypertensive treatment for patients with acute ischemic stroke with or without history of hypertension: a secondary analysis of the CATIS randomized clinical trial. JAMA Netw Open. 2019;2(7):e198103. 31365109 10.1001/jamanetworkopen.2019.8103 PMC6669782

[28] Al‐Mashhadi RH , Al‐Mashhadi AL , Nasr ZP , et al. Local pressure drives low‐density lipoprotein accumulation and coronary atherosclerosis in hypertensive minipigs. J Am Coll Cardiol. 2021;77(5):575‐589. 33538256 10.1016/j.jacc.2020.11.059

[29] Feng X , Chan KL , Lan L , et al. Translesional pressure gradient alters relationship between blood pressure and recurrent stroke in intracranial stenosis. Stroke. 2020;51(6):1862‐1864. 32312220 10.1161/STROKEAHA.119.028616

[30] Raghuram K , Durgam A , Kohlnhofer J , Singh A . Relationship between stroke recurrence, infarct pattern, and vascular distribution in patients with symptomatic intracranial stenosis. J Neurointerv Surg. 2018;10(12):1161‐1163. 29602861 10.1136/neurintsurg-2017-013735

[31] Lovett JK , Howard SC , Rothwell PM . Pulse pressure is independently associated with carotid plaque ulceration. J Hypertens. 2003;21(9):1669‐1676. 12923399 10.1097/00004872-200309000-00016

[32] Wen HM , Lam WW , Rainer T , et al. Multiple acute cerebral infarcts on diffusion‐weighted imaging and risk of recurrent stroke. Neurology. 2004;63(7):1317‐1319. 15477564 10.1212/01.wnl.0000140490.22251.b6

[33] Jing J , Suo Y , Wang A , et al. Imaging Parameters predict recurrence after transient ischemic attack or minor stroke stratified by ABCD(2) score. Stroke. 2021;52(6):2007‐2015. 33947206 10.1161/STROKEAHA.120.032424

[34] Group SPSS , Benavente OR , Coffey CS , et al. Blood‐pressure targets in patients with recent lacunar stroke: the SPS3 randomised trial. Lancet. 2013;382(9891):507‐515. 23726159 10.1016/S0140-6736(13)60852-1 PMC3979302

[35] He J , Zhang Y , Xu T , et al. Effects of immediate blood pressure reduction on death and major disability in patients with acute ischemic stroke: the CATIS randomized clinical trial. JAMA. 2014;311(5):479‐489. 24240777 10.1001/jama.2013.282543

