# Supplementary material for: Systolic blood pressure and recurrent stroke in patients with different lesion patterns on diffusion weighted imaging
Source: J Clin Hypertens (Greenwich). 2022 Aug 12;24(10):1350–7. doi: 10.1111/jch.14543 (PMC9581100; doi:10.1111/jch.14543)
Supplement: Supplementary file 1 — Supporting Information. [file JCH-24-1350-s001.docx]

Supplemental table Ⅰ. Baseline characteristics of patients included vs not included in this substudy of the CHANCE trial.

|  | Patients Included  (N=1089) | All Others in CHANCE  (N=4081) | *P* Value |
| --- | --- | --- | --- |
| Age, mean ± SD, y | 63.2 ± 10.7 | 62.5 ± 10.7 | 0.0658 |
| Male, n (%) | 713 (65.5) | 2707 (66.3) | 0.5946 |
| Current/previous smoking, n (%) | 458 (42.1) | 1763 (43.2) | 0.4983 |
| Current/ previous drinking, n (%) | 335 (30.8) | 1265 (31.0) | 0.8814 |
| Body mass index, mean ± SD, kg/m^2^ | 24.4 ± 3.2 | 24.7 ± 3.0 | 0.0021 |
| Admission NIHSS score, median (IQR) | 2.0 (0.0-2.0) | 1.0 (0.0-2.0) | 0.0051 |
| Medical history, n (%) |  |  |  |
| Ischemic stroke/TIA | 213 (19.6) | 961 (23.5) | 0.0052 |
| Myocardial infarction | 19 (1.7) | 77 (1.9) | 0.7577 |
| Atrial fibrillation | 21 (1.9) | 75 (1.8) | 0.8440 |
| Heart Failure | 19 (1.7) | 61 (1.5) | 0.5526 |
| Hypertension | 710 (65.2) | 2689 (65.9) | 0.6684 |
| Diabetes mellitus | 227 (20.8) | 866 (21.2) | 0.7875 |
| Hyperlipidemia | 92 (11.4) | 481 (11.0) | 0.7350 |
| Antiplatelet treatment |  |  | 0.7975 |
| Clopidogrel + Aspirin | 531 (48.8) | 2053 (50.3) |  |
| Aspirin only | 558 (51.2) | 2028 (49.7) |  |

Abbreviations: CHANCE, Clopidogrel in High-Risk Patients with Acute Nondisabling Cerebrovascular Events; SD, standard deviation; NIHSS, National Institute of Health Stroke Scale; IQR, interquartile range; TIA, transient ischemic attack.

Supplemental table Ⅱ. Effects of baseline systolic blood pressure on 90-day efficacy and safety outcomes based on stroke subtypes.

| **Subgroup**  Outcome | No. of Patients | Crude Event Rates No. (%) | | Crude | | Multivariable Adjusted | |
| --- | --- | --- | --- | --- | --- | --- | --- |
|  |  | SBP<140mmHg | SBP ≥140mmHg | HR (95% CI) | *P* Value | HR (95% CI) | *P* Value |
| **Non-Lacunar Infarctione** | 439 |  |  |  |  |  |  |
| Stroke Recurrence |  | 10 (9.6) | 54 (16.1) | 1.70 (0.87-3.34) | 0.2900 | 1.55 (0.78-3.10) | 0.2109 |
| Combined vascular events |  | 10 (9.6) | 55 (16.4) | 1.73 (0.88-3.40) | 0.1093 | 1.57 (0.79-3.14) | 0.1971 |
| Bleeding |  | 1 (1.0) | 6 (1.8) | 1.58 (0.18-13.55) | 0.6749 | 6.07 (0.32-114.6) | 0.2291 |
| **Lacunar Infarction** | 392 |  |  |  |  |  |  |
| Stroke Recurrence |  | 2 (2.7) | 19 (6.0) | 2.20 (0.51-9.43) | 0.2900 | 2.46 (0.56-10.74) | 0.2318 |
| Combined vascular events |  | 2 (2.7) | 19 (6.0) | 2.20 (0.51-9.43) | 0.2900 | 2.46 (0.56-10.74) | 0.2318 |
| Bleeding |  | 2 (2.7) | 9 (2.8) | 1.03 (0.22-4.77) | 0.9701 | 0.99 (0.21-4.78) | 0.9922 |
| **Negative DW Imaging** | 258 |  |  |  |  |  |  |
| Stroke Recurrence |  | 2 (2.5) | 6 (3.4) | 1.37 (0.28-6.80) | 0.6976 | 1.17 (0.22-6.28) | 0.8572 |
| Combined vascular events |  | 3 (3.7) | 6 (3.4) | 0.91 (0.23-3.65) | 0.8974 | 0.73 (0.17-3.10) | 0.6682 |
| Bleeding |  | 1 (1.2) | 4 (2.3) | 1.85 (0.21-16.55) | 0.5824 | 1.46 (0.15-14.16) | 0.7439 |

Abbreviations: HR, hazard ratio; CI, confidence interval; DWI, diffusion weighted imaging.

Adjusted for age, gender, body mass index, current or previous smoking, medical history of stroke, TIA, hypertension and hyperlipidemia treatment, and randomization group.
